# Prognostic marker and target in prostate cancer

**DOI:** 10.18632/aging.100827

**Published:** 2015-10-20

**Authors:** Giovanni Lavorgna, Francesco Montorsi, Andrea Salonia

**Affiliations:** Division of Oncology/Unit of Urology, URI, IRCCS Ospedale San Raffaele, Milan, Italy

**Keywords:** ncRNA, biomarker, therapeutic target, apoptosis, prostate cancer

In physiological conditions, Reactive Oxigen Species (ROS) are present in low amounts and their action can be effectively counteracted by the antioxidant system of the cells. However, cancer cells have higher ROS levels as a consequence of their increased metabolic activity, leading to a state of chronic oxidative stress. Increased levels of ROS are indeed involved in tumorigenesis and progression, conferring a growth advantage to malignant cells thanks to the upregulation of pathways stimulating proliferation and maintenance of the tumoral phenotype. In keeping with this point, cancer cells have a well-adapted antioxidant system to balance their increased generation of ROS [1]. Nevertheless, excess oxidative stress due to increase of ROS levels beyond a certain threshold can become harmful for cancer cells by damaging macromolecules vital for cellular function. Along this line, therapeutic approaches aimed at increasing oxidative stress by producing a further increase in ROS levels of tumor cells have been proposed [1]. In this scenario, it is likely that the activity of molecules like TRPM2, an ion channel member of the transient receptor potential (TRP) superfamily, that is able to mediate cell death upon increased oxidative stress [2], needs to be kept under a tight control in malignant cells as well. Thanks to a computational effort [3], we identified a few years ago TRPM2-AS, an antisense, non-coding RNA transcript mapped within the genomic locus of human TRPM2 [4]. TRPM2-AS appeared to be over-expressed in several tumor types and, given the possible negative regulatory role of antisense transcripts on their sense partner [5], we reasoned that its putative function in cancer cells was to regulate the activity of the TRPM2 gene under conditions of elevated oxidative stress. However, since basal levels of TRPM2 are still required in cancer cells [6], its role was more likely to prevent over-activation of the channel rather than completely abolishing its function.

Recently, our group evaluated the role of TRPM2-AS in prostate cancer (PCa) [7]. We investigated first TRPM2-AS expression levels and found that it was overexpressed both in tumor specimens and tumor cell lines in respect to their healthy counterpart. Moreover, its expression was found to be correlated with poor prognosis indicators, like the presence of metastases, higher Gleason score, higher PSA, positive lymph nodes, biochemical relapse, etc. We also derived a gene signature associated to high expression levels of TRPM2-AS in patients. Remarkably, this signature robustly predicted the disease outcome in three independent patient coohorts, including patients with low Gleason score. Ablation of TRPM2-AS expression in PCa cells by RNAi led to massive apoptosis both in *in-vitro* and *in-vivo* experiments, demonstrating the essential tole of this ncRNA for survival of PCa cells. Expression profiling of targeted cells showed an unbearable increase of oxidative stress in the dying cells, coupled to an intracellular increase of hydrogen peroxide and activation of the sense TRPM2 gene [7]. Taken together, these data hint to the role of TRPM2-AS in keeping in check both the oxidative stress and the activity of the ion channel TRPM2 in PCa cells. It is intriguing to note that several recent sequencing projects, including the The Cancer Genome Atlas (http://cancergenome.nih.gov/), have failed to show frequent inactivating mutations in the TRPM2 gene in PCa patients and in other tumor types (data not shown). This is in agreement with the result of RNAi experiments indicating that basal TRPM2 levels are still required for survival of PCa cells [6]. Therefore, it is conceivable that modulation of the activity of this channel is achieved by epigenetic means or by coexpression of regulatory transcripts like TRPM2-AS. Intriguingly, other TRPM2 smaller isoforms, working as dominant negative, have been found in bone marrow [2] and in several tumor types [4], suggesting that the fine tuning of TRPM2 expression might be even more complex than anticipated.

From a translational point of view, our data support the role of TRPM2-AS as a novel prognostic marker and therapeutic target in PCa. TRPM2-AS levels, indeed, could be used to identify PCa patients that are likely to have a poor prognosis and that are in need of treatment. Among the therapeutic options, treatments aimed at increasing cellular oxidative stress should be considered. Patient selection should occur upon gene expression analysis of the biopsy sample. However, since recent reports have shown that it is possible to quantify ncRNAs in exosomes from urine even without digital rectal examination [8], application of this approach should, in principle, allow a minimally invasive measurement of TRPM2-AS in PCa patients. It should also be considered that, given the recent improvements both in the design and delivery of siRNAs and antisense oligonucleotides, TRPM2-AS itself could be exploited as a druggable therapeutic target in the near future.
